# Trunk Orientation, Stability, and Quadrupedalism

**DOI:** 10.3389/fneur.2013.00020

**Published:** 2013-03-14

**Authors:** Y. P. Ivanenko, W. G. Wright, R. J. St George, V. S. Gurfinkel

**Affiliations:** ^1^Laboratory of Neuromotor Physiology, Santa Lucia FoundationRome, Italy; ^2^Department of Physical Therapy and Department of Bioengineering, Temple UniversityPhiladelphia, PA, USA; ^3^Department of Neurology, Oregon Health and Science UniversityPortland, OR, USA

**Keywords:** locomotion, bipedalism and quadrupedalism, muscle tone, posture control, gait pathology, humans

## Abstract

Interesting cases of human quadrupedalism described by Tan and Colleagues (2005–2012) have attracted the attention of geneticists, neurologists, and anthropologists. Since his first publications in 2005, the main attention has focused on the genetic aspects of disorders that lead to quadrupedalism within an evolutionary framework. In recent years this area has undergone a convincing critique (Downey, 2010) and ended with a call “… to move in a different direction … away from thinking solely in terms of genetic abnormality and evolutionary atavism.” We consider quadrupedalism as a “natural experiment” that may contribute to our knowledge of the physiological mechanisms underlying our balance system and our tendency toward normal (upright) posture. Bipedalism necessitates a number of characteristics that distinguish us from our ancestors and present-day mammals, including: size and shape of the bones of the foot, structure of the axial and proximal musculature, and the orientation of the human body and head. In this review we address the results of experimental studies on the mechanisms that stabilize the body in healthy people, as well as how these mechanisms may be disturbed in various forms of clinical pathology. These disturbances are related primarily to automatic rather than voluntary control of posture and suggest that human quadrupedalism is a behavior that can result from adaptive processes triggered by disorders in postural tone and environmental cues. These results will serve as a starting point for comparing and contrasting bi- and quadrupedalism.

## Introduction

Recent publications discussing the phenomenon of “quadrupedal humans” have attracted attention not only due to the rarity of this form of disease, but more controversially due to suggestions that this syndrome should be considered an example of the regression of the evolutionary process, or a syndrome of “human devolution” or “reverse evolution.” On this matter a lively debate has arisen (Tan, 2005, 2006, 2008; Humphrey et al., 2008; Downey, 2010; Karaca et al., 2012). The phenomena, described as “a rare natural experiment” (for review, see Tan, 2010), are of interest not only for geneticists and evolutionists, but also for physiologists and clinicians examining posture and balance problems. Unfortunately, the postural aspect in these papers is presented rather parsimoniously, however some impressions can be drawn based on the video content (e.g., Tan and Tan, 2010,^1^). For instance, it is evident that these patients have difficulty maintaining a familiar orthograde posture and balance, and that they use an unusual form of quadrupedalism. But these data serve as illustrations and have not undergone rigorous biokinematic analysis (measurement of angular and linear displacement, velocity, acceleration, etc.). An additional limitation to interpreting the results from these case studies is that they include only reports of neurological symptoms and do not contain information describing the state of the musculoskeletal system (muscle strength, range of motion in the joints, muscle tone). Without such information, these publications remain intriguing observations.

Analysis of these cases, using the full arsenal of tools that are applied in studies of locomotion and balance would reveal the mechanisms underlying neurophysiological adaptations to specific gaits and postures. For a description of the characteristics of quadrupedal gait in healthy human infants and adults we refer readers to other reviews (Prost, 1980; Falgairolle et al., 2006; Dietz and Michel, 2009; Patrick et al., 2009, 2012; Zehr et al., 2009; Gallagher et al., 2011; Zampagni et al., 2011; MacLellan et al., 2012; Thibaudier and Hurteau, 2012). In this paper, we will focus on the mechanisms stabilizing the body as a starting point for comparing bipedalism and quadrupedalism.

## Posture and Bipedalism

Let us consider the characteristics of human bipedalism but not confine ourselves to its dictionary definition of “movement performed only on two legs,” since there are numerous attributes, that must be considered to gain a full appreciation of bipedalism. Indeed, clinical evidence points toward an important role of the mechanisms determining a specific body configuration or posture.

The basis of habitual human posture (e.g., sitting or standing) is *postural tone* of the skeletal muscles. Postural tone is an unconscious, low-amplitude, long-lasting muscle tension distributed in a specific pattern along the entire body axis. Differences in postural alignments and facial expressions across individuals are determined by this small but specific active muscle tone (Gurfinkel et al., 1995, 2006). Reduced or absent facial expression (i.e., “poker face”) in Parkinson’s Disease (Jankovic, 2003) may provide a useful example of how a pathological increase in tone can affect facial musculature. Furthermore, changes in the state of this tonic muscle activity have been shown experimentally to affect postural orientation (Kluzik et al., 2005; Wright, 2011) as well as gait parameters (Mori et al., 1982; Ivanenko et al., 2006; Selionov et al., 2009). As Sherrington (1906) so accurately expresses in the statement “posture follows movement like a shadow,” this highlights the interrelation of posture and movement since movement is not possible without appropriate tonic activity and disturbances of postural tone may therefore affect the whole body movement. Mori et al. (1978, 1982) provided very convincing experimental evidence of this when they showed that tone is of crucial importance for gait. They demonstrated that, in both the mesencephalic and intact cat, brainstem evoked locomotion must be preceded by an increase in postural muscle tone.

The inability to adopt a natural, upright pose might be thought of as a disruption of the habitual distribution of postural tone. Therefore it is essential to have an idea of how postural tone is generated and how it is controlled. Regarding this question, there exist very basic descriptions of processes formed mainly from animal experimentation. In the most recent reviews on this topic (e.g., Pérennou, 2012), we find involvement of the stretch reflex (considering the role of Ia and II afferents), positive reaction of the support, vestibular, and tonic neck reflexes, crossed extensor reflexes, role of vision, reference frames, anticipatory mechanisms, body scheme, contact with the support, role of spinal, and supraspinal structures, neuromodulators, and many other factors contributing to the distribution of postural tone. But how does this all result in an appropriate posture? Although the muscle mechanics such as twitch, and fused and unfused tetanus have been well-characterized after more than a century of research, how these mechanisms are used to drive postural tone are not clearly understood. Instead, we typically see only schemes, diagrams, or models which attempt to integrate them. More often what shapes our understanding of postural tone comes from clinical observations, which provide a rich data set of behavioral outcomes, despite not allowing us to directly examine the underlying mechanisms. Nevertheless, clinical cases about disturbances of postural tone, shed light on these schemes and help adapt the models with regard to how postural tone affects postural orientation in humans. The problem proves particularly challenging in the case of bipedalism, an inherently unstable posture, which would require constant conscious attention to maintain, if not for the contribution of unconscious, tonic control. Therefore, the effects of disturbances to postural tone on postural orientation must be considered if we wish to understand disturbances to bipedalism.

## Pathological Disturbances of Postural Tone

**Figure 1:**
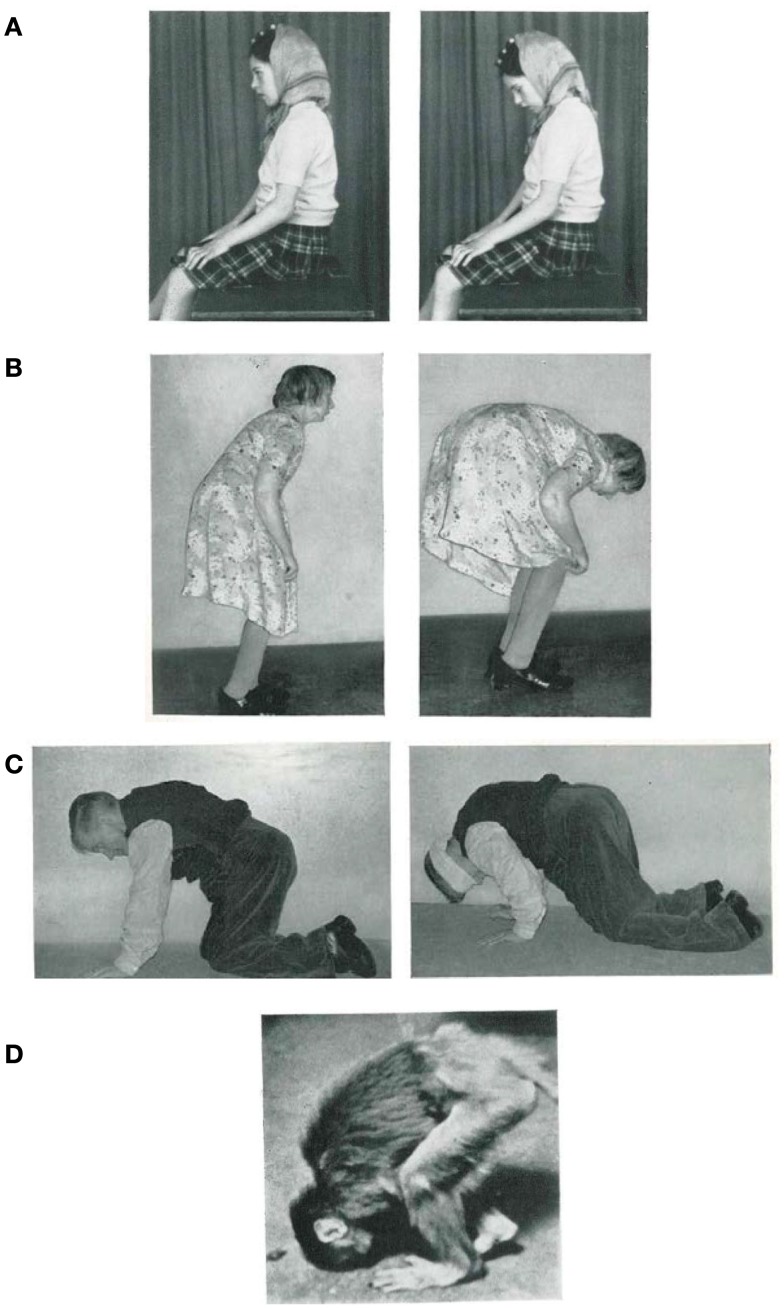
**(A)** Postural fixation of the head is impaired at all times (*left*), but as soon as the patient closes her eyes her head falls forward until her chin rests on her chest (*right*). **(B)** Patient standing as erect as she can (*left*), and 2 or 3 min later (*right*). **(C)** Patient’s usual position when on all fours (*left*), and 1 min after blindfolding (*right*). **(D)** “Flexed posture” in a monkey with severe bilateral degeneration of the *Globus pallidus* [adapted from Martin (1967), with permission].

The memorable findings obtained by the British neurologist James Purdon Martin, described in his famous book “The Basal Ganglia and Posture” (1967) provide excellent examples of how disturbances to postural tone affect the ability to maintain natural upright stance, which we argue in this review to be an integral part of bipedalism. A first example that we draw from Martin is the case of a little girl with Wilson’s disease whose head was observed to tilt down whenever she closed her eyes. As soon as she reopened them, her head orientation automatically returned to normal again (Figure 1A). Despite this behavior, she was quite capable even with her eyes closed to raise her head when asked to do so, and even push back against strong resistance. Many other similar examples of adult patients are reported by Martin, with one particularly illustrative case being a patient who showed loss of normal posture of the head and trunk when in the sitting position with closed eyes. In this case study Martin reports on an older adult woman who is unable to hold her body up and gradually becomes more flexed at the hips as she walks (Figure 1B). It is notable that the slope of the body increases with increasing back kyphosis. From the descriptions of these two patients one important aspect can be stressed: these patients were able to maintain a familiar upright pose, if they focused attention on their posture and used voluntary control to perform the task. Another example taken from Martin’s investigations shows a patient who cannot actively maintain his posture when on all fours (Figure 1C). Initially, he is able to hold his head horizontally, but once blindfolded, the head falls forward. Though the dropping of the head tended to be a gradual process over a few minutes, if performing an additional task such as crawling, the head could be seen to drop abruptly.

To explain all these cases, Martin introduced the concept of “postural fixation,” which he defines as, “the fixation or support of a segment of the body on adjoining segments, or on the body as a whole, for postural purposes.” Although he provides “postural fixation” as a descriptive expression only, making no assertions regarding its physiological basis (see p. 9, Martin, 1967), it still provides a helpful conceptual framework with which to view posture and gait together. Martin proposes the above examples are the result of a loss of fixation activity of the neck muscles, back muscles, or even the entire body. In some of the cases reporting the loss of fixation activity of back muscles, the patient may be reduced to moving on all fours until able to regain an upright posture without focused effort. Although this last case seems to be evidence for a loss of bipedalism, what can be said about the first two examples (Figures 1A,B)? Can we assume that they are also related to the disorders of bipedalism? Is there a qualitative difference between them? If not, then what is human bipedalism?

On this subject there are differing definitions, however, we favor the one given by the renowned paleoanthropologist Phillip Tobias (1925–2012): “*Human uprightness is among the most striking characteristics that distinguish living man from the great apes of Africa and Asia. Uprightness of the body has two aspects to it: erectness of the trunk and two-footed, or bipedal, stance and gait. It would be reasonable to suppose that a well-developed sense of balance must have been a prerequisite to, or at least an accompaniment of, the development of uprightness*” (Tobias, 1992). In this definition the two most striking characteristics of human uprightness have been delineated: a vertical trunk upon two feet. Other features of the vertical posture of man are: the vaulted structure of the feet (Wright et al., 2012), the relative length of the legs, specificity of the knee, and hip joints, the inside oblique position of the thigh bones, the position and structure of the pelvis and chest, the S-shaped curvature of the spine, and the structure and orientation of the skull. This myriad list of features related to upright posture suggests that violations of bipedalism may take many different forms.

**Figure 2:**
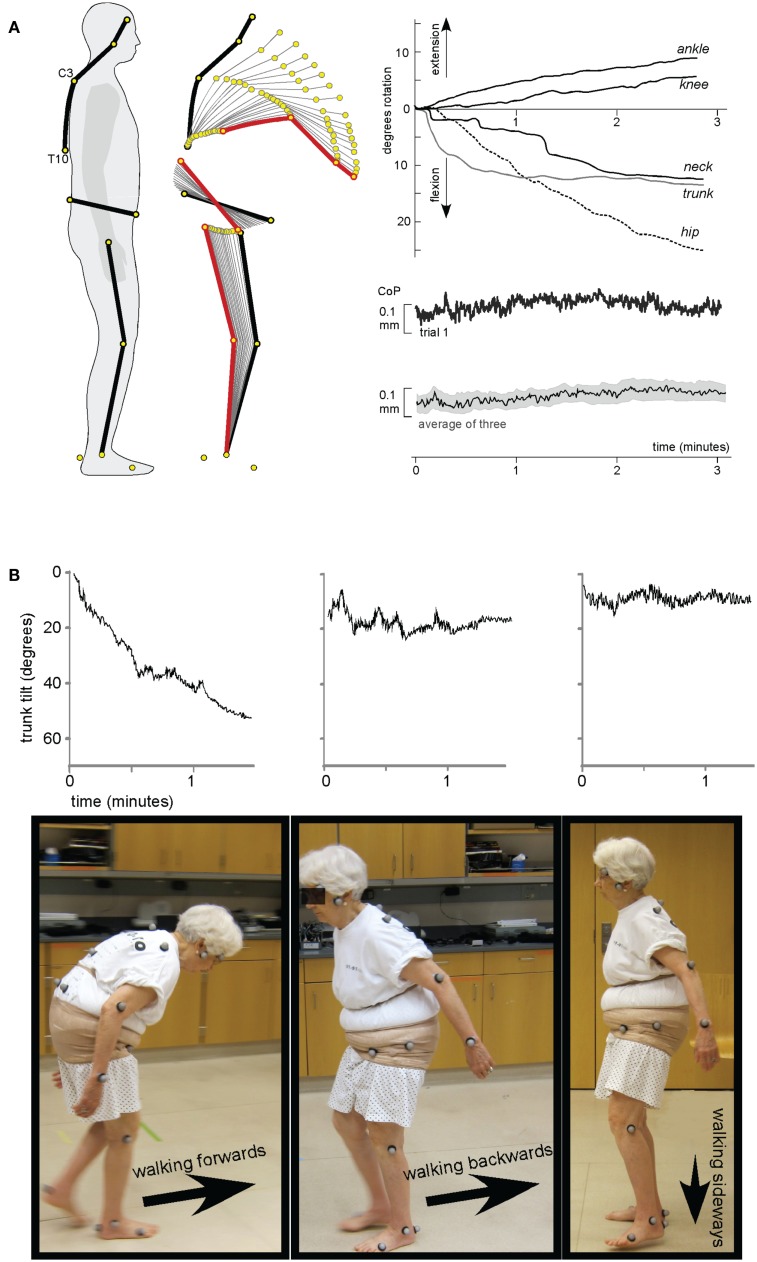
**A case of Camptocormia**. **(A)** A kinematic study of the time-course of the involuntary forward bending immediately following arising from a chair reveals a profound loss of postural alignment, however, balance control is intact. The segments of the body move in a coordinated way that ensures that the center of pressure remains steady over the base of support. **(B)** Trunk flexion when walking forward, but NOT when walking backward or sideways [the data were adapted from St George et al. (2010)].

The study of these features can provide an additional source of information facilitating understanding of the physiological mechanisms for human bipedalism. Disturbances of postural tone have also been documented in animals (see, for instance, monkey, Figure 1D; Richter, 1945), but we can also provide a similar example in humans. We restrict ourselves to just one example from our experience (St George et al., 2010) encountered in an 82-year-old woman who had had idiopathic camptocormia [derived from the Greek words: *kamptos* (to bend) and *kormos* (trunk)] for 20 years but was otherwise healthy. She reported no family history of similar disorders and her neurological status was otherwise without abnormality. When specialized postural tests were performed (e.g., perturbation of the standing surface, bilateral Achilles tendon vibration), she showed normal automatic postural responses and intact voluntary control of postural musculature. Perceptually the patient could correctly indicate the subjective vertical and horizontal. During quiet stance (Figure 2A) the trunk consistently flexed forward, initially through the spine, followed by the pelvis. The flexion followed an exponential decay with a time constant of 34 s (SD = 5 s) and after 2 min the upper trunk was relatively stable at 83° (SD = 8) from vertical. Despite this trunk flexed position and the relative change of all body segments, the center of pressure remained stable beneath the two feet. This appears to be due to a synergistic extension of the ankle and knee joints resulting in a backward shift in the pelvis. Gait analysis showed trunk flexion and drooping arms in forward walking. Notably, while this patient walks bipedally with the trunk flexed, when ascending a staircase she prefers to walk on all fours. In contrast, during walking backward or sideways the trunk remained erect (Figure 2B). This difference in posture during backward and sideways walking may occur because these two forms of locomotion are less automatic than forward walking.

This example is of special interest to us in that it illustrates the following three concepts: (I) in the regulation of human posture two different control systems participate – a system that provides appropriate positioning of the body segments (and thus entire posture) and a system that provides balance of the body, (II) that some categories of patients with disorders of posture are able to stand and walk upright, if they concentrate on doing so, and (III) posture and gait disturbances are related primarily to an automatic rather than voluntary regime of their control.

## Concluding Remarks on Human Quadrupedalism

Upright bipedal walking is one of the most highly automated motor acts that adults perform. The human bipedal gait and heel-to-toe rolling pattern are unique (Bramble and Lieberman, 2004) and require a specific inter-segmental coordination, motor patterns, balance control, and walking experience for acquisition of plantigrade gait at the beginning of independent walking (Forssberg, 1985; Ivanenko et al., 2007; Dominici et al., 2011; Lacquaniti et al., 2012). Nevertheless, we can crawl and perform “stoopwalking,” both of which are compulsory gait techniques in some occupational settings, e.g., in a low-seam coal mine (Gallagher et al., 2011). Skilled climbing, another form of quadrupedalism, is also within the human repertoire of locomotion (Gebo, 1996; Zampagni et al., 2011). On the other hand, anyone who has ever been to the circus knows many quadrupedal animals can be trained to stand and walk bipedally. Thus, quadrupedalism and bipedalism in animals, and in humans in particular, can be both habitual and temporary. The question that remains, though, is what are the principles that determine habitual gait?

Simplified explanations arising from gene abnormalities may not necessarily be helpful for the detailed investigation of the principles underlying the manifestation of human quadrupedalism. The genetic program indeed determines a general sequence of maturation of the central nervous system and musculoskeletal apparatus, although it has one characteristic feature – critical developmental windows for the transition. Such time periods are well known in relation to a number of other sensorimotor systems. The expression “critical period” in the context of the developing mammalian visual system was introduced by the groundbreaking work of Wiesel and Hubel (1963). Humans and songbirds have similar critical periods for vocal learning (Doupe and Kuhl, 1999). A critical postnatal period can be defined as a developmental window during which specific experiences have a greater effect than at other times. If this window is missed there cannot be a full return to the normal developmental process. Among various examples, one can mention feral children who have acquired quadrupedal gait at an early age and conserved its characteristics (see, for instance, the most recent case of the Ukrainian girl^2^. If one takes into account all these descriptions, infants who came to live with animals very early (i.e., prior to acquiring bipedal gait), had substantial difficulties in learning to stand and walk bipedally after returning to the human community. Those whose time with animals came later (such as the above-mentioned Ukrainian girl), adapted back to upright walking more easily. Even though most later learn to walk bipedally, in cases of emergency they have been found to automatically switch to the previously acquired quadrupedal gait. The review of Hensch (2004) summarizes our current understanding of known critical periods across several systems and species, demonstrating that appropriate critical period development is a prerequisite to proper motor control and coordinated movement later in life.

The emergence of human quadrupedalism is thought to have a number of interacting causes including physical factors [e.g., cerebellar impairment, vestibular impairment, cognitive impairment, polio-induced weakness in the legs, and dynamic instability during upright walking, in addition to psychological and social factors (Tan, 2010)]. Taking into account the facility with which the central nervous system is able to adapt when faced with a specific gait pathology, often it is difficult to distinguish what primarily comes from pathology and what comes from compensatory mechanisms. We suggest that many adaptive features in individuals with Uner Tan syndrome are likely compensatory, including quadrupedal gait. In various reported cases of human quadrupedalism, an erect posture can be voluntarily maintained although the preferred posture and gait are quadrupedal (Tan and Tan, 2010). Thus, we would like to stress again that these disturbances are related primarily to automatic rather than voluntary control of upright posture and suggest that human quadrupedalism is a behavior that may result from adaptive processes triggered by disorders in postural tone. Tonogenic circuitry is a component of several, diverse supraspinal structures, including rostral ward, for example, the reticular formation, vestibular nuclei, the cerebellum, and selected mesodiencephalic nuclei (Hess, 1954). Degradation of postural tone may also affect quadrupedal gait and other motor activities, moreover the transition to functional quadrupedal gait may involve a set of additional challenges. Nevertheless, as we discussed in the previous section, the control of postural *stability* may or may not be related to the mechanisms underlying the control of postural *tone*, depending on pathology, which fits well with the observations that individuals with Uner Tan syndrome are typically able to voluntarily maintain an upright posture. In our view, any reflection on the nature of human quadrupedalism should include a consideration of the mechanisms determining the choice of unconscious habitual posture.

## Conflict of Interest Statement

The authors declare that the research was conducted in the absence of any commercial or financial relationships that could be construed as a potential conflict of interest.

## Footnotes

^1^http://www.webmedcentral.com/article_view/831

^2^http://www.youtube.com/watch?v=qyqbnDjId7g
